# Supplementary material for: Nicotine dependence among critically ill COVID-19 patients: A population-based cohort study
Source: PLoS One. 2026 Apr 22;21(4):e0308776. doi: 10.1371/journal.pone.0308776 (PMC13102216; doi:10.1371/journal.pone.0308776)
Supplement: S1 Table — (PDF) [file pone.0308776.s001.pdf]

S1 Table. ICD-10-CM codes

S1 Table. *International Classification of Diseases, Tenth Revisions, Clinical Modification* (ICD-10-CM) codes	
**Diagnosis**	**ICD-10-CM**
COVID-19	U071
Current nicotine dependence	F17200, F17208, F17209, F17210, F17218, F17219, F17220, F17228, F17229, F17290, F17298, F17299,
Former nicotine dependence	Z87891, F17201, F17203, F17211, F17213, F17221, F17223, F17291, F17293
Palliative care	Z515
Do not resuscitate	Z66
