# Supplementary material for: Nicotine dependence among critically ill COVID-19 patients: A population-based cohort study
Source: PLoS One. 2026 Apr 22;21(4):e0308776. doi: 10.1371/journal.pone.0308776 (PMC13102216; doi:10.1371/journal.pone.0308776)
Supplement: S2 Table — (PDF) [file pone.0308776.s002.pdf]

S2 Table. The distribution of nicotine dependence and tobacco use among critically-ill COVID-19 patients

S2 Table. The distribution of nicotine and tobacco diagnoses among critically-ill COVID-19 patients		
**Nicotine and tobacco diagnoses**	**ICD-10-CM[a] codes**	**Count (%)**
		n = 142,045
**Nicotine dependence, unspecified**		
Any	F1720x	2,825 (2.0)
Uncomplicated	F17200	2,796 (2.0)
In remission	F17201	21 (0.0)
With withdrawal	F17203	8 (0.0)
With other nicotine-induced disorders	F17208	1 (0.0)
With unspecified nicotine-induced disorders	F17209	0 (0.0)
**Nicotine dependence, cigarettes**		
Any	F1721x	6,729 (4.7)
Uncomplicated	F17210	6,648 (4.7)
In remission	F17211	59 (0.0)
With withdrawal	F17213	16 (0.0)
With other nicotine-induced disorders	F17218	2 (0.0)
With unspecified nicotine-induced disorders	F17219	5 (0.0)
**Nicotine dependence, chewing tobacco**		
Any	F1722x	347 (0.2)
Uncomplicated	F17220	345 (0.2)
In remission	F17221	2 (0.0)
With withdrawal	F17223	0 (0.0)
With other nicotine-induced disorders	F17228	0 (0.0)
With unspecified nicotine-induced disorders	F17229	0 (0.0)
**Nicotine dependence, other tobacco product**		
Any	F1729x	836 (0.6)
Uncomplicated	F17290	835 (0.6)
In remission	F17291	0 (0.0)
With withdrawal	F17293	0 (0.0)
With other nicotine-induced disorders	F17298	0 (0.0)
With unspecified nicotine-induced disorders	F17299	1 (0.0)
**Personal history of nicotine dependence**	Z87891	23,601 (16.6)
**Other tobacco and nicotine codes**		

Tobacco use not otherwise specified	Z720	700 (0.5)
Tobacco abuse counseling	Z716	539 (0.4)
Contact with and (suspected) exposure to environmental tobacco smoke (acute) (chronic)	Z7722	294 (0.2)
Occupational exposure to environmental tobacco smoke	Z5731	6 (0.0)
Family history of tobacco abuse and dependence	Z812	3 (0.0)
Toxic effect of tobacco and nicotine	T652x	2 (0.0)
Tobacco use disorder complicating pregnancy, childbirth, and the puerperium	O9933x	0 (0.0)
Newborn affected by maternal use of tobacco	P042	0 (0.0)

[a] *ICD-10-CM: International Classification of Diseases, Tenth Revisions, Clinical Modification*
