# Supplementary material for: Nicotine dependence among critically ill COVID-19 patients: A population-based cohort study
Source: PLoS One. 2026 Apr 22;21(4):e0308776. doi: 10.1371/journal.pone.0308776 (PMC13102216; doi:10.1371/journal.pone.0308776)
Supplement: S3 Table — (PDF) [file pone.0308776.s003.pdf]

S3 Table. Variance inflation factors

S3 Table. Variance inflation factors			
**Variable**	**GVIF**[a]	**DF**[b]	$\sqrt[2\,DF]{GVIF}$
Nicotine status	1.1	1	1.0
Age	2.0	2	1.2
Sex	2.5	2	1.3
Race/ethnicity	1.4	3	1.1
Insurance	1.9	5	1.1
Number of organ dysfunctions	1.3	1	1.1
Deyo comorbidity index	9.0	1	3.0
Myocardial infarction	1.1	1	1.1
Peripheral vascular disease	1.1	1	1.0
Dementia	1.6	1	1.3
Peptic ulcer disease	1.0	1	1.0
Hemiplegia / Paraplegia	1.2	1	1.1
Human immunodeficiency virus	1.3	1	1.1
Congestive heart failure	2.9	1	1.7
Cerebrovascular disease	1.1	1	1.1
Renal disease	2.3	1	1.5
Lung disease	1.7	1	1.3
Rheumatological disease	1.1	1	1.1
Chronic liver disease	1.8	1	1.3
Diabetes	1.2	1	1.1
Any malignancy	2.4	1	1.6
Mental disorders	1.1	1	1.1
Malnutrition	1.1	1	1.0
Obesity	1.1	1	1.1
Alcohol use	1.6	1	1.3
Drug use	1.5	1	1.2
Hemodialysis	1.3	1	1.2
Invasive mechanical ventilation	1.2	1	1.1
Blood transfusion	1.1	1	1.1
Do not resuscitate	1.5	1	1.2
Palliative care	1.3	1	1.1
Teaching hospital	1.1	1	1.0
Year	1.3	4	1.0

[a] GVIF: Generalized variance inflation factor

[b] DF: Degrees of freedom
