# Supplementary material for: Nicotine dependence among critically ill COVID-19 patients: A population-based cohort study
Source: PLoS One. 2026 Apr 22;21(4):e0308776. doi: 10.1371/journal.pone.0308776 (PMC13102216; doi:10.1371/journal.pone.0308776)
Supplement: S4 Table — (PDF) [file pone.0308776.s004.pdf]

S4 Table. Alternative modeling for potential impact of multicollinearity

S4 Table. Alternative modeling for the potential impact of multicollinearity

Model	Currently dependent	Never dependent	aRR [95% CI][a]	aRD [95% CI][b]
	mortalities total no. (%)			
**With Deyo comorbidity index**				
In-hospital mortality	1,115 / 10,452 (10.7)	17,385 / 107,922 (16.1)	0.8955 [0.8572 to 0.9356]	-0.0134 [-0.0185 to -0.0083]
Short-term mortality	1,410 / 10,452 (13.5)	21,618 / 107,922 (20.0)	0.8926 [0.8618 to 0.9245]	-0.0174 [-0.0225 to -0.0122]
**Without Deyo comorbidity index**				
In-hospital mortality	1,115 / 10,452 (10.7)	17,385 / 107,922 (16.1)	0.8960 [0.8577 to 0.9361]	-0.0134 [-0.0185 to -0.0083]
Short-term mortality	1,410 / 10,452 (13.5)	21,618 / 107,922 (20.0)	0.8934 [0.8626 to 0.9253]	-0.0172 [-0.0224 to -0.0121]

[a] aRR [95% CI]: Adjusted risk ratio and 95% confidence interval

[b] aRD [95% CI]: Adjusted risk difference and 95% confidence interval
