# Supplementary material for: Nicotine dependence among critically ill COVID-19 patients: A population-based cohort study
Source: PLoS One. 2026 Apr 22;21(4):e0308776. doi: 10.1371/journal.pone.0308776 (PMC13102216; doi:10.1371/journal.pone.0308776)
Supplement: S5 Table — (PDF) [file pone.0308776.s005.pdf]

S5 Table. Sensitivity analyses for type of current nicotine dependence or tobacco use

S5 Table. Sensitivity analyses by types of dependence or tobacco use for association with mortality among critically-ill COVID-19 patients.

Exposure group	Exposure	Never dependent[a]	aRR [95% CI][b]	aRD [95% CI][c]	*p* value
	mortalities / total no. (%)				
**Current nicotine dependence, unspecified**					
In-hospital mortality	298 / 2,747 (10.8)	17,303 / 107,230 (16.1)	0.8446 [0.7780 to 0.9170]	-0.0203 [-0.0294 to -0.0111]	0.0001
Short-term mortality	378 / 2,747 (13.8)	21,514 / 107,230 (20.1)	0.8810 [0.8261 to 0.9396]	-0.0192 [-0.0284 to -0.0100]	0.0001
**Current nicotine dependence, cigarettes**					
In-hospital mortality	663 / 6,478 (10.2)	17,303 / 107,230 (16.1)	0.8934 [0.8442 to 0.9456]	-0.0131 [-0.0194 to -0.0068]	0.0001
Short-term mortality	855 / 6,478 (13.2)	21,514 / 107,230 (20.1)	0.8904 [0.8510 to 0.9316]	-0.0173 [-0.0237 to -0.0108]	< 0.0001
**Current nicotine dependence, chewing tobacco**					
In-hospital mortality	46 / 321 (14.3)	17,303 / 107,230 (16.1)	0.8906 [0.7375 to 1.0756]	-0.0181 [-0.0461 to 0.0100]	0.2290
Short-term mortality	49 / 321 (15.3)	21,514 / 107,230 (20.1)	0.8048 [0.6811 to 0.9508]	-0.0380 [-0.0645 to -0.0114]	0.0107
**Current nicotine dependence, other tobacco product**					
In-hospital mortality	90 / 723 (12.4)	17,303 / 107,230 (16.1)	1.0014 [0.8756 to 1.1453]	0.0002 [-0.0165 to 0.0169]	0.9832
Short-term mortality	105 / 723 (14.5)	21,514 / 107,230 (20.1)	0.9455 [0.8447 to 1.0584]	-0.0084 [-0.0248 to 0.0080]	0.3301
**Tobacco use not otherwise specified**					
In-hospital mortality	82 / 692 (11.8)	17,303 / 107,230 (16.1)	0.9625 [0.8350 to 1.1095]	-0.0047 [-0.0217 to 0.0124]	0.5980
Short-term mortality	104 / 692 (15.0)	21,514 / 107,230 (20.1)	1.0033 [0.8989 to 1.1198]	0.0005 [-0.0163 to 0.0173]	0.9531

[a] Excludes hospitalizations with tobacco use not otherwise specified

[b] aRR [95% CI]: Adjusted risk ratio and 95% confidence interval

[c] aRD [95% CI]: Adjusted risk difference and 95% confidence interval
