# Supplementary material for: Nicotine dependence among critically ill COVID-19 patients: A population-based cohort study
Source: PLoS One. 2026 Apr 22;21(4):e0308776. doi: 10.1371/journal.pone.0308776 (PMC13102216; doi:10.1371/journal.pone.0308776)
Supplement: S6 Table — (PDF) [file pone.0308776.s006.pdf]

S6 Table. Exploratory analyses for evaluation of potential selection bias

S6 Table. Exploratory analyses for the evaluation of potential selection bias				
**Exposure**	**Exposed**	**Unexposed**	**aRR [95% CI][a]**	***p* value**
	mortalities / total no. (%)			
**Current nicotine dependence**				
In-hospital mortality	1,115 / 10,452 (10.7)	17,385 / 107,922 (16.1)	0.8955 [0.8572 to 0.9356]	< 0.0001
Short-term mortality	1,410 / 10,452 (13.5)	21,618 / 107,922 (20.0)	0.8926 [0.8618 to 0.9245]	< 0.0001
**Chronic lung disease**				
In-hospital mortality	3,468 / 23,540 (14.7)	15,032 / 94,834 (15.9)	0.9832 [0.9512 to 1.0162]	0.3138
Short-term mortality	4,628 / 23,540 (19.7)	18,400 / 94,834 (19.4)	0.9865 [0.9622 to 1.0114]	0.2857
**Congestive heart failure**				
In-hospital mortality	4,557 / 23,837 (19.1)	13,943 / 94,537 (14.7)	1.0256 [0.9783 to 1.0752]	0.2937
Short-term mortality	6,031 / 23,837 (25.3)	16,997 / 94,537 (18.0)	1.0031 [0.9679 to 1.0396]	0.8658
**Malignancy**				
In-hospital mortality	880 / 4,777 (18.4)	17,620 / 113,597 (15.5)	0.9762 [0.8561 to 1.1131]	0.7190
Short-term mortality	1,274 / 4,777 (26.7)	21,754 / 113,597 (19.2)	1.0545 [0.9632 to 1.1544]	0.2510
**Diabetes**				
In-hospital mortality	9,000 / 51,703 (17.4)	9,500 / 66,671 (14.2)	0.9899 [0.9695 to 1.0108]	0.3406
Short-term mortality	10,889 / 51,703 (21.1)	12,139 / 66,671 (18.2)	0.9738 [0.9577 to 0.9901]	0.0017
**Obesity**				
In-hospital mortality	6,770 / 39,788 (17.0)	11,730 / 78,586 (14.9)	1.0818 [1.0596 to 1.1046]	< 0.0001
Short-term mortality	7,599 / 39,788 (19.1)	15,429 / 78,586 (19.6)	1.0369 [1.0194 to 1.0548]	< 0.0001
**Alcohol abuse**				
In-hospital mortality	321 / 1,881 (17.1)	18,179 / 116,493 (15.6)	0.9216 [0.8285 to 1.0252]	0.1330
Short-term mortality	393 / 1,881 (20.9)	22,635 / 116,493 (19.4)	0.9482 [0.8730 to 1.0298]	0.2068
**Drug abuse**				
In-hospital mortality	350 / 2,224 (15.7)	18,150 / 116,150 (15.6)	0.8889 [0.8151 to 0.9694]	0.0078
Short-term mortality	410 / 2,224 (18.4)	22,618 / 116,150 (19.5)	0.8716 [0.8123 to 0.9352]	0.0001
**Mental disorders**				

In-hospital mortality	4,617 / 25,239 (18.3)	13,883 / 93,135 (14.9)	1.0043 [0.9817 to 1.0274]	0.7130
Short-term mortality	5,873 / 25,239 (23.3)	17,155 / 93,135 (18.4)	0.9998 [0.9824 to 1.0175]	0.9806

[a] aRR [95% CI]: Adjusted risk ratio and 95% confidence interval
