# Supplementary material for: Nicotine dependence among critically ill COVID-19 patients: A population-based cohort study
Source: PLoS One. 2026 Apr 22;21(4):e0308776. doi: 10.1371/journal.pone.0308776 (PMC13102216; doi:10.1371/journal.pone.0308776)
Supplement: S7 Table — (PDF) [file pone.0308776.s007.pdf]

S7 Table. Non association of covariates with nicotine dependence in the overlap weighted population

S7 Table. Non association of covariates with nicotine dependence in the overlap weighted population			
**Variables**	**Logistic regression coefficient**	**Standard error**	***p* value**
Intercept	0.00000000	0.00000000	0.99969050
Age 45 to 64 years	0.00000000	0.00000000	0.99998516
Age ≥ 65 years	0.00000000	0.00000000	0.99993745
Male	0.00000000	0.00000000	0.99963118
Suppressed sex data	0.00000000	0.00000000	0.99995664
Hispanic	0.00000000	0.00000000	0.99960845
Black	0.00000000	0.00000000	0.99991679
Other race	0.00000000	0.00000000	0.99994010
Medicare	0.00000000	0.00000000	0.99997170
Medicaid	0.00000000	0.00000000	0.99979625
other insurance	0.00000000	0.00000000	0.99976724
Uninsured	0.00000000	0.00000000	0.99975308
Missing insurance data	0.00000000	0.00000001	0.99998572
Total organ dysfunctions	0.00000000	0.00000000	0.99982126
Deyo index	0.00000000	0.00000000	0.99999651
Deyo MI	0.00000000	0.00000000	0.99971974
Deyo PVD	0.00000000	0.00000000	0.99972712
Dementia	0.00000000	0.00000000	0.99993321
Peptic ulcer disease	0.00000000	0.00000000	0.99999103
Hemiplegia / paraplegia	0.00000000	0.00000000	0.99995605
Human immunodeficiency virus	0.00000000	0.00000001	0.99998055
Congestive heart failure	0.00000000	0.00000000	0.99999944
Cerebrovascular disease	0.00000000	0.00000000	0.99998433
Renal disease	0.00000000	0.00000000	0.99995664
Chronic lung disease	0.00000000	0.00000000	0.99978638
Rheumatological disease	0.00000000	0.00000000	0.99998385
Chronic liver disease	0.00000000	0.00000000	0.99998781
Diabetes	0.00000000	0.00000000	0.99988888
Malignancy	0.00000000	0.00000000	0.99999899

Mental disorders	0.00000000	0.00000000	0.99980810
Malnutrition	0.00000000	0.00000000	0.99994821
Obesity	0.00000000	0.00000000	0.99965503
Alcohol abuse	0.00000000	0.00000000	0.99998775
Drug abuse	0.00000000	0.00000000	0.99998733
Hemodialysis	0.00000000	0.00000000	0.99996736
Invasive mechanical ventilation	0.00000000	0.00000000	0.99972450
Blood transfusion	0.00000000	0.00000000	0.99993686
Do not resuscitate	0.00000000	0.00000000	0.99983907
Palliative care	0.00000000	0.00000000	0.99983115
Teaching hospital	0.00000000	0.00000000	0.99999190
Year2021	0.00000000	0.00000000	0.99977662
Year2022	0.00000000	0.00000000	0.99947277
Year2023	0.00000000	0.00000000	0.99950317
Year2024	0.00000000	0.00000000	0.99986920
