# Supplementary figures and images for: Nicotine dependence among critically ill COVID-19 patients: A population-based cohort study
Source: PLoS One. 2026 Apr 22;21(4):e0308776. doi: 10.1371/journal.pone.0308776 (PMC13102216; doi:10.1371/journal.pone.0308776)

S1 Fig. Density plots of propensity scores

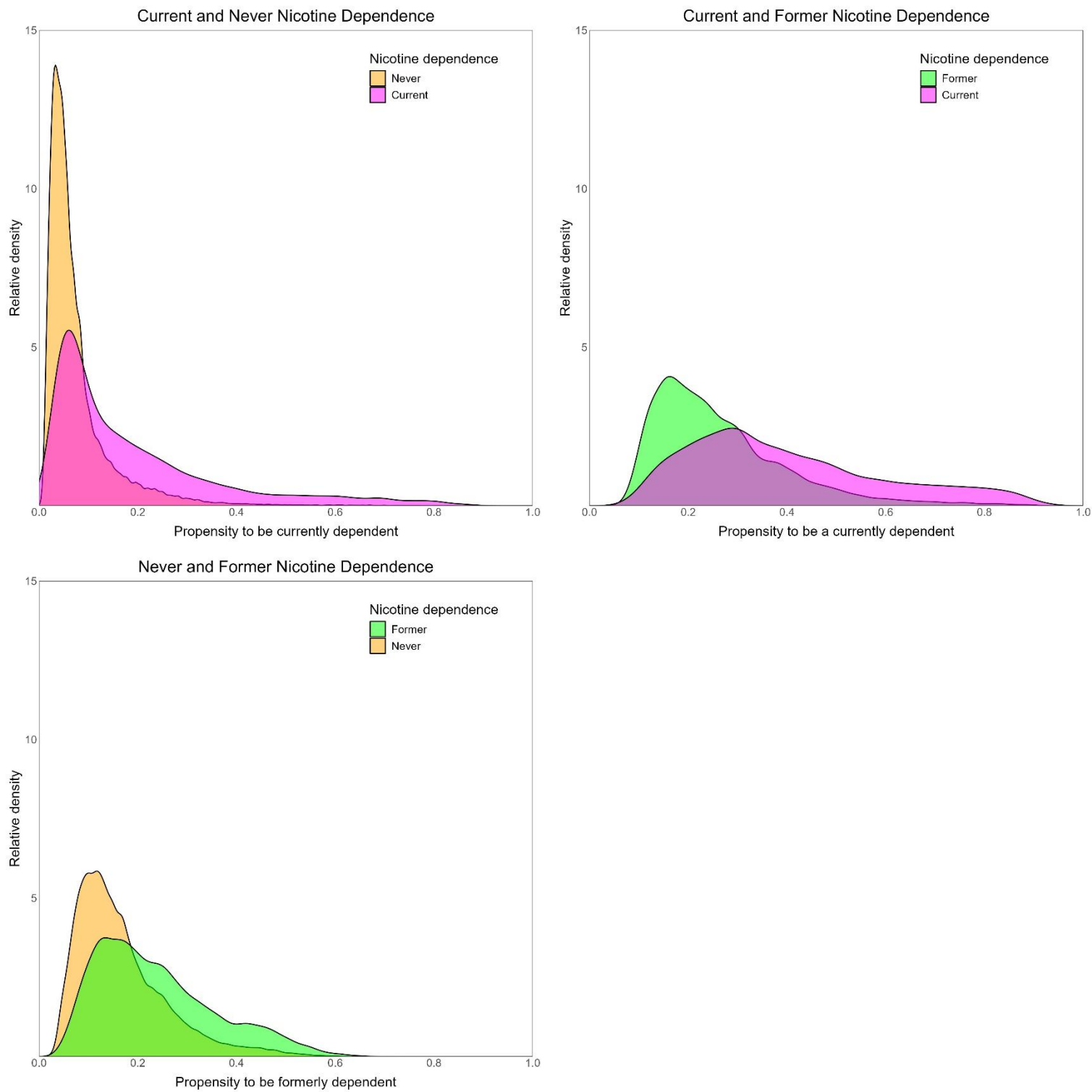

Supplement: S1 Fig — (PDF) [file pone.0308776.s008.pdf]
