# Supplementary material for: Nicotine dependence among critically ill COVID-19 patients: A population-based cohort study
Source: PLoS One. 2026 Apr 22;21(4):e0308776. doi: 10.1371/journal.pone.0308776 (PMC13102216; doi:10.1371/journal.pone.0308776)
Supplement: S1 File — (PDF) [file pone.0308776.s009.pdf]

S1 File. R code.

setup

```
# packages
library(tidyverse)
library(moderndive)
library(magrittr)
library(knitr)
library(matlib)
library(readxl)
library(readr)
library(writexl)
library(icdpicr)
library(PSweight)
library(beepr)
library(lme4)
library(ebal)
library(aweSOM)
library(tidyverse)
library(ggplot2)
library(nnet)
library(broom)
library(knitr)
library(kableExtra)
library(gtsummary)
library(ggeffects)
library(marginaleffects)
library(car)
library(nnet)
library(WeightIt)

# options
knitr::opts_chunk$set(
    eval       = FALSE,
    echo       = TRUE,
    error      = FALSE,
    fig_align  = 'center',
    fig_height = 8,
    fig_width  = 8,
    message    = FALSE,
    warning    = FALSE,
    comment    = NULL,
    include    = TRUE,
    prompt     = FALSE)

# clear
remove(list = ls())

# directory
directory <- '~/covid/revised_analyses'
setwd(directory)
```

import_all

```
# directory
setwd(directory)

# import
M <- read_rds('data_ready.rds')
```

create_subsets

```
# directory
setwd(directory)

# filter
Ma <- filter(M, nicotine_status %in% c('current', 'never'))
Mb <- filter(M, nicotine_status %in% c('current', 'former'))
Mc <- filter(M, nicotine_status %in% c('former', 'never'))

# reference_levels
Ma$nicotine_status <- relevel(Ma$nicotine_status, ref = 'never')
Mb$nicotine_status <- relevel(Mb$nicotine_status, ref = 'former')
Mc$nicotine_status <- relevel(Mc$nicotine_status, ref = 'never')

# save
write_rds(
  x        = Ma,
  file     = 'data_a.rds',
  compress = 'gz')

write_rds(
  x        = Mb,
  file     = 'data_b.rds',
  compress = 'gz')

write_rds(
  x        = Mc,
  file     = 'data_c.rds',
  compress = 'gz')
```

import_subset

```
# clear_environment
remove(list = ls())

# directory
setwd(directory)

# import
M <- read_rds('data_a.rds')
M %<>% mutate(nicotine_status = droplevels(nicotine_status))

# subgroup
group <- 'all'

# response
```

```
response <- c('hospital_mortality', 'm_h')[1]

# mutate
M$response <- M[[response]]
```

overlap_weights

```
# propensity_formula
formula_ps <-
  # exposure
  nicotine_status ~
  # covariates
  age +
  sex_missing +
  race_ethnicity +
  insurance +
  oftot +
  deyoind +
  deyomi +
  deyopvd +
  deyodem +
  deyopud +
  deyohemi +
  deyohiv +
  deyochf +
  deyocvd +
  deyockd +
  deyolung +
  deyorheum +
  chrliver +
  dm +
  malign +
  comment +
  commalnut +
  comobes1 +
  comalc2 +
  comdrg3 +
  prochd +
  procimvall +
  procbld +
  dnr +
  palcare +
  teaching_hosp +
  year


# overlap_weights
WT <-
  weightit(
    formula     = formula_ps,
    data        = M,
    method      = 'glm',
    estimand    = 'ATO',
    stabilize   = FALSE,
    focal       = NULL,
    by          = NULL,
    s.weights   = NULL,
    ps          = NULL,
```

```
    moments     = NULL,
    int         = FALSE,
    subclass    = NULL,
    missing     = NULL,
    verbose     = FALSE,
    include.obj = FALSE,
    keep.mparts = TRUE)
```

g_computation

```
# formula_out
 <-
  # response
  response ~
  # exposure
  nicotine_status +
  # covariates
  age +
  sex_missing +
  race_ethnicity +
  insurance +
  oftot +
  deyoind +
  deyomi +
  deyopvd +
  deyodem +
  deyopud +
  deyohemi +
  deyohiv +
  deyochf +
  deyocvd +
  deyockd +
  deyolung +
  deyorheum +
  chrliver +
  dm +
  malign +
  comment +
  commalnut +
  comobes1 +
  comalc2 +
  comdrg3 +
  prochd +
  procimvall +
  procbld +
  dnr +
  palcare +
  teaching_hosp +
  year

# weighted_regression
model_out <-
  glm_weightit(
    formula    = formula_out,
    data       = M,
    family     = binomial,
    weightit   = WT,
```

```
    vcov      = 'asympt',
    cluster   = NULL,
    R         = 500L,
    offset    = NULL,
    start     = NULL,
    x         = FALSE,
    y         = TRUE,
    contrasts = NULL,
    fwb.args  = list(),
    br        = FALSE)

# risk_ratio
RR <-
  avg_comparisons(
    model       = model_out,
    newdata     = M,
    variables   = 'nicotine_status',
    type        = NULL,
    vcov        = TRUE,
    by          = TRUE,
    conf_level  = 0.95,
    comparison  = 'lnratioavg',
    transform   = 'exp',
    cross       = FALSE,
    wts         = WT$weights,
    hypothesis  = NULL,
    equivalence = NULL,
    df          = Inf,
    eps         = NULL,
    numderiv    = 'fdforward')

# risk_difference
RD <-
    avg_comparisons(
      model       = model_out,
      newdata     = M,
      variables   = 'nicotine_status',
      type        = NULL,
      vcov        = TRUE,
      by          = TRUE,
      conf_level  = 0.95,
      comparison  = 'difference',
      transform   = NULL,
      cross       = FALSE,
      wts         = WT$weights,
      hypothesis  = NULL,
      equivalence = NULL,
      df          = Inf,
      eps         = NULL,
      numderiv    = 'fdforward')
```

clinical_equipoise_a

```
# formula_out
formula_e <-
  # response
```

```r
  nicotine_status ~
  # covariates
  age +
  sex_missing +
  race_ethnicity +
  insurance +
  oftot +
  deyoind +
  deyomi +
  deyopvd +
  deyodem +
  deyopud +
  deyohemi +
  deyohiv +
  deyochf +
  deyocvd +
  deyockd +
  deyolung +
  deyorheum +
  chrliver +
  dm +
  malign +
  comment +
  commalnut +
  comobes1 +
  comalc +
  comdrg +
  prochd +
  procimvall +
  procbld +
  dnr +
  palcare +
  teaching_hosp +
  year

# weighted_regression
model_e <-
  glm_weightit(
    formula   = formula_e,
    data      = M,
    family    = binomial,
    weightit  = WT,
    vcov      = 'asymp',
    cluster   = NULL,
    R         = 500L,
    offset    = NULL,
    start     = NULL,
    x         = FALSE,
    y         = TRUE,
    contrasts = NULL,
    fwb.args  = list(),
    br        = FALSE)

# summary
w <- summary(model_e)

# coefficients
w <- w[['coefficients']]
```

```
# row_names
rnames <- rownames(w)

# tibble
w %<>% as.tibble

# format
w %<>%
  mutate(
    Estimate = round(Estimate, digits = 8),
    Estimate = format(Estimate, nsmall = 8, scientific = FALSE))

# names
names(w) <- c('estimate', 'std_error', 'z_value', 'p_value')
w %<>% mutate(vars = rnames)

# format
w %<>%
  mutate(
    std_error = round(std_error, digits = 8),
    std_error = format(std_error, nsmall = 8, scientific = FALSE))

# format
w %<>%
  mutate(
    p_value = round(p_value, digits = 8),
    p_value = format(p_value, nsmall = 8, scientific = FALSE))

# deselect
w %<>% dplyr::select(-3)

# save
write_xlsx(
  x              = w,
  path           = 'table_e.xlsx',
  col_names      = TRUE,
  format_headers = TRUE,
  use_zip64      = FALSE)
```

clinical_equipoise_b

```
# predict
probs <-
  predict(
    object  = model_e,
    newdata = M,
    type    = 'response')

# format
probs <- round(probs, digits = 8)
probs <- format(probs, nsmall = 8, scientific = FALSE)

# table
table(probs)
```

```
probs
0.50000000
    118372
```

simple_bootstraps

```
f <- function(i){

# sample
index <- sample(1:nrow(M), size = nrow(M), replace = TRUE)
SM    <- M[index,]

# filter
SM %<>% filter(race_ethnicity != 'missing')
SM %<>% mutate(race_ethnicity = droplevels(as.factor(race_ethnicity)))

# propensity_model
model_ps <-
  glm(
    formula = formula_ps,
    data    = SM,
    family  = 'binomial')

# propensity_scores
propensity <-
  predict(
    object  = model_ps,
    newdata = SM,
    type    = 'response')

# overlap_weights
SM$propensity <- propensity

SM$overlap <-
  ifelse(
    test = SM$nicotine_status == 'never',
    yes  = SM$propensity,
    no   = 1 - propensity)

# normalize_weights
SM$wts <- SM$overlap / sum(SM$overlap)

# outcome_model
model_out <-
  glm(
    formula = formula_out,
    data    = SM,
    weights = SM$wts,
    family  = 'binomial')

# counter_factuals
SM0 <- mutate(SM, nicotine_status = 'never')
SM1 <- mutate(SM, nicotine_status = 'current')

# predictions
preds_0 <-
```

```r
  predict(
    object  = model_out,
    newdata = SM0,
    type    = 'response')

preds_1 <-
  predict(
    object  = model_out,
    newdata = SM1,
    type    = 'response')

# average_potential_outcomes
mu_0 <- sum(SM0$wts * preds_0) / sum(SM0$wts)
mu_1 <- sum(SM1$wts * preds_1) / sum(SM1$wts)

# marginal_effects
rr <- mu_1 / mu_0
rd <- mu_1 - mu_0

# tibble
tibble(
  rr = rr,
  rd = rd)}

# list_apply
v <- lapply(1:10000, 'f')

# bind_rows
vv <- bind_rows(v)
```

confidence_interval

```r
# sort
vv <-
  mutate(
    vv,
    rr = sort(rr),
    rd = sort(rd))

# pull
rr <- vv[['rr']]
rd <- vv[['rd']]

# bounds
lwr <- rr[25]
upr <- rr[975]

# display
lwr;upr

[1] 0.8520752

[1] 0.9307676

# ci_width
upr - lwr
```

```
[1] 0.07869234

# primary_method_table_three

0.9356 - 0.8572

[1] 0.0784
```

effective_sample_size

```
# clear_environment
remove(list = ls())

# directory
directory <- '~/covid_three/revised_analysis'
setwd(directory)

# import
M <- read_rds('data_a.rds')
M %<>% mutate(nicotine_status = droplevels(nicotine_status))

# response
response <- c('hospital_mortality', 'm_h')[1]

# transform
M$response <- M[[response]]
M$response <- as.numeric(M$response)

# filter
M %<>% filter(race_ethnicity != 'missing')
M %<>% mutate(race_ethnicity = droplevels(as.factor(race_ethnicity)))

# propensity_formula
formula_ps  <-
  # exposure
  nicotine_status ~
  # covariates
  age +
  sex_missing +
  race_ethnicity +
  insurance +
  oftot +
  deyoind +
  deyomi +
  deyopvd +
  deyodem +
  deyopud +
  deyohemi +
  deyohiv +
  deyochf +
  deyocvd +
  deyockd +
  deyolung +
  deyorheum +
  chrliver +
  dm +
  malign +
  comment +
```

```r
  commalnut +
  comobes1 +
  comalc +
  comdrg +
  prochd +
  procimvall +
  procbld +
  dnr +
  palcare +
  teaching_hosp +
  year

# propensity_model
model_ps <-
  glm(
    formula = formula_ps,
    data    = M,
    family  = 'binomial')

# propensity_score
propensity <-
  predict(
    object  = model_ps,
    newdata = M,
    type    = 'response')

# transform
M$propensity <- propensity

# overlap_weights
M$overlap <-
  ifelse(
    test = M$nicotine_status == 'never',
    yes  = M$propensity,
    no   = 1 - propensity)

# subgroups
M0 <- filter(M, nicotine_status == 'never')
M1 <- filter(M, nicotine_status == 'current')

# effective_sample_sizes
ess0 <- (sum(M0$overlap)) ^ 2 / sum((M0$overlap) ^ 2)
ess1 <- (sum(M1$overlap)) ^ 2 / sum((M1$overlap) ^ 2)

# never_dependent
nrow(M0);ess0

[1] 107920

[1] 54722.82

# current_dependent
nrow(M1);ess1

[1] 10452

[1] 9978.929
```
