# Supplementary material for: Nicotine dependence among critically ill COVID-19 patients: A population-based cohort study
Source: PLoS One. 2026 Apr 22;21(4):e0308776. doi: 10.1371/journal.pone.0308776 (PMC13102216; doi:10.1371/journal.pone.0308776)
Supplement: Attachment — Submitted filename: Response to Reviewers.pdf [file pone.0308776.s011.pdf]

Dear PLOS ONE,

Thank you for the reviewer comments regarding the submission:

"A paradoxical outcome of smokers among hospitalized patients with COVID-19: A population-based cohort study" (PONE-D-24-31829).

A point-by-point response to the reviewer comments is provided below.

- "The manuscript contains several grammatical errors."

We have corrected many grammatical errors and have carefully reviewed the manuscript for grammatical and stylistic improvements. For example, the abstract of the attached file "Revised Manuscript with Track Changes" has new text highlighted in green and deletions in red with strikethrough.

- "The Introduction is weak and needs improvement."

The introduction has been redone. There is a different emphasis with new references added. We have attempted to make the introduction align with the scale and scope of similar manuscripts on related topics.

- "Furthermore, the Discussion relies heavily on potential mechanisms from other studies rather than focusing on the interpretation of the current study's findings. A strong Discussion should integrate data from other studies while primarily emphasizing the interpretation of the authors' own results. Currently, there is an overemphasis on external studies and insufficient interpretation and discussion of the authors' own work."

After considering the reviewers comments, our research team decided to remove the original discussion of potential mechanisms from the manuscript. Although we believe hypotheses regarding potential mechanisms are important and interesting, we also believe that the previous discussion is better suited for a separate research project and is out of place here.

We have divided a new discussion section into two subsections. The new "key findings" subsection adds additional commentary on tables one through four that we believe will aid readers in better interpreting the study results. A new "relationship to previous studies" section highlights the differences in design and conclusions between our study and previous studies on the topic. We believe it is worth mentioning that a large and diverse volume of research has been published on this topic from authors spanning the globe, making it impractical to mention all relevant studies.

John Garza

John Garza, PhD
