# Supplementary material for: Nicotine dependence among critically ill COVID-19 patients: A population-based cohort study
Source: PLoS One. 2026 Apr 22;21(4):e0308776. doi: 10.1371/journal.pone.0308776 (PMC13102216; doi:10.1371/journal.pone.0308776)
Supplement: Attachment — Submitted filename: Response_to_Reviewers_auresp_2.pdf [file pone.0308776.s012.pdf]

Dear PLOS ONE,

Thank you for the reviewer comments regarding the submission (PONE-D-24-31829):

"A paradoxical outcome of smokers among hospitalized patients with COVID-19: A population-based cohort study"

A point-by-point response to the reviewer comments is provided below.

- Reviewer #2: The authors have made changes as per the previous reviewer recommendations. It is an interesting study with a large patient population. Their results are different than many of the earlier observational and meta-analysis looking at smoking and COVID-19. This would have given an opportunity to have an interesting discussion and highlight their results and how they got strikingly different results. The attached discussion in the revised manuscript still lacks that and they discuss no more than 2-3 studies. I strongly suggest the authors to have an extensive discussion highlighting other meta-analysis and why do their results differ. An entire page on discussion is just summarizing the results.

  Like for example- Some large-scale registry-based studies have concluded that current smokers have similar risk of in-hospital mortality compared to nonsmokers- No reference attached. How did these studies differ than theirs and why do they have different results- is it because of race, gender, age or geographical location?

  Other registry-based studies have in contrast concluded that smokers are at increased risk of in-hospital mortality. Using
  data from a voluntary American Heart Association hospital data registry, Poudel et al found smokers to be at increased risk of in-hospital mortality. Just mentioning results is not discussion- they need to then further elaborate why are their results different and how we should infer this study.

We thank the reviewer for these important comments.

We have increased the number of previous studies considered to fifteen.

We emphasize differences in the definitions of smoking, mortality, cohort, and time periods of the different studies.

Within the methods and materials section, we now explain how our exposure variable should be interpreted in relation to those used in other studies.

We state that differences in parameters make many previous studies incomparable to ours.

We emphasize the characteristics of our study that make it a meaningful contribution to what is known on the topic.

In the conclusions section, we conclude that a decisive consensus on the association of smoking with mortality requires additional research.

John Garza

John Garza, PhD
