# Supplementary material for: Nicotine dependence among critically ill COVID-19 patients: A population-based cohort study
Source: PLoS One. 2026 Apr 22;21(4):e0308776. doi: 10.1371/journal.pone.0308776 (PMC13102216; doi:10.1371/journal.pone.0308776)
Supplement: Attachment — Submitted filename: Response_to_Reviewers_auresp_3.pdf [file pone.0308776.s013.pdf]

September 10, 2025

Dear PLOSONE,

We appreciate the many suggestions that have been provided and hope the valuable reviewer comments have led to enhanced rigor, interpretability, and overall quality. We have extended the study period through Q2 of 2024 as this data became available during the revisions process. We now have three exposure groups and have used overlap weights to create the overlap population at clinical *equipoise*. We apply weighted logistic regression and g-computation for risk-adjusted analysis on the overlap population. Due to the reviewer comments, we have realized the importance of adding do-not-resuscitate orders and palliative care as risk adjustment variables. We have added an exploratory analysis for potential selection bias, conducted sensitivity analysis for unmeasured confounders using E-Values, and provided lower bounds on selection bias needed to explain away the result. In response to reviewer questions and recommendations, we have refined our cohort derivation. Because of these many changes the methods and materials section is new. The results section has also been redone to match the new analyses procedure. The introduction section is the same as before and the discussion has only been revised, enhanced, clarified in response to the reviewers' recommendations and questions and is essentially along the same lines as before. Despite the revised analysis procedure and cohort derivation, the conclusions of the study remain as before. Despite the many enhancements and clarifications there were a few reviewer recommendations that we could not satisfy and a few reviewer questions we could not answer. We respectfully request a pardon for the inability to satisfy all the recommendations and answer all the questions.

Sincerely,

John Garza

John Garza, PhD

Associate Professor

Texas Tech University School of Medicine

gar31703@ttuhsc.edu

(512) 350-1444

Major Concerns:

1. Study Cohort Definition and Indication for Hospitalization

• The manuscript states that the study includes hospitalized patients with a diagnosis of COVID-19 (ICD-10-CM U07.1 in any diagnosis column). This means that the cohort includes both patients admitted due to COVID-19 and those hospitalized for other reasons who happened to have a COVID-19 diagnosis. This significantly affects the interpretation of the results. Patients who were hospitalized due to COVID-19 may have different mortality risks than those hospitalized for another condition (e.g., cancer, heart failure) but tested positive for COVID-19. Thus, smoking associations with COVID-19 related mortality can be skewed.

• While the authors attempted to adjust for this by including a sensitivity analysis limited to patients with a principal diagnosis of COVID-19, this approach does not fully resolve potential confounding.

• Recommendation: The authors should clarify in discussion how this affects the results and could explain the results of this study.

We appreciate the reviewers' important observations and valuable recommendations. We have changed the cohort to only involve patients with a principal diagnosis of COVID-19. This can be observed in the new Figure 2: Cohort derivation.

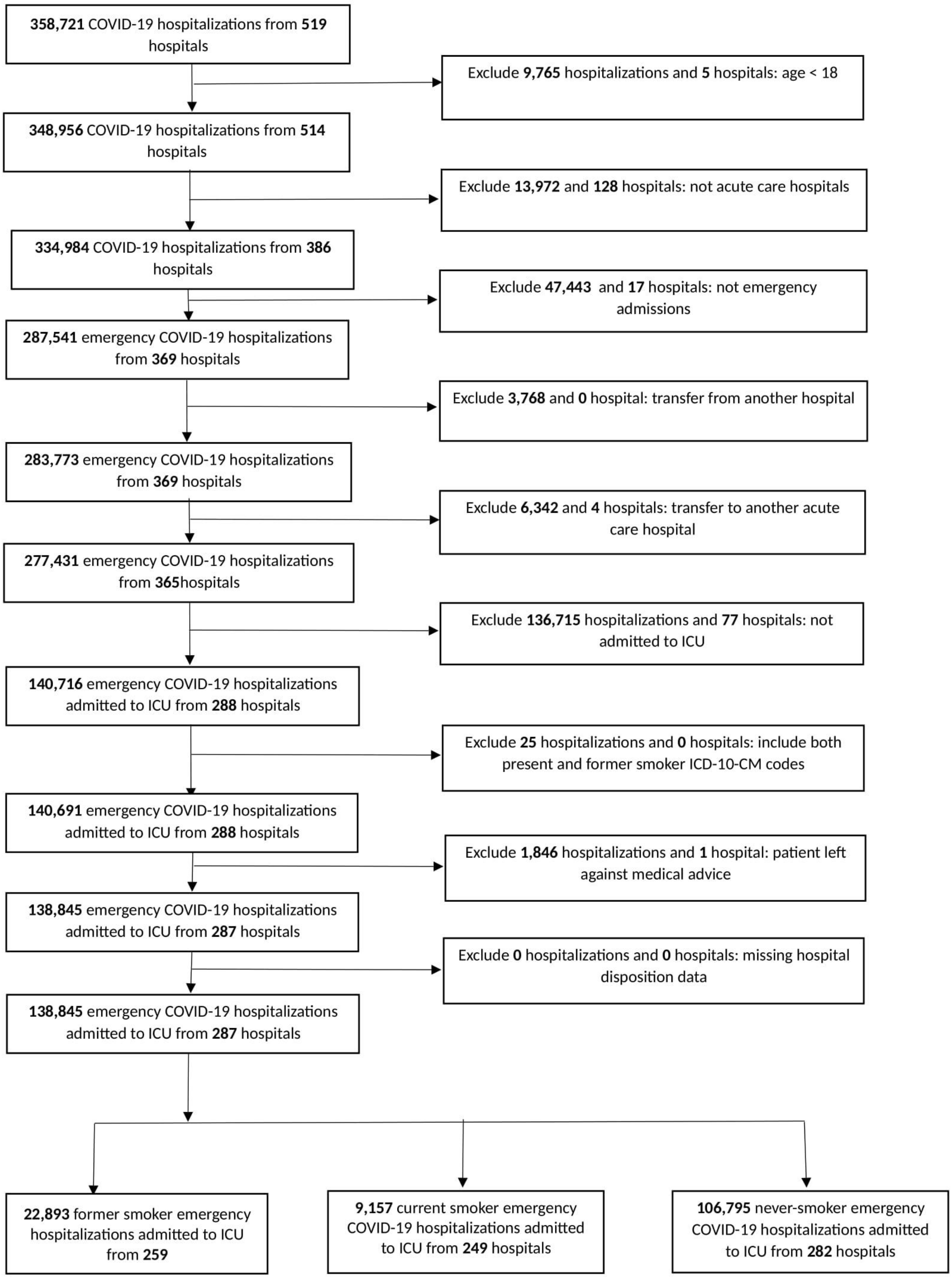

358,721 COVID-19 hospitalizations from 519 hospitals
Exclude 9,765 hospitalizations and 5 hospitals: age < 18
348,956 COVID-19 hospitalizations from 514 hospitals
Exclude 13,972 and 128 hospitals: not acute care hospitals
334,984 COVID-19 hospitalizations from 386 hospitals
Exclude 47,443 and 17 hospitals: not emergency admissions
287,541 emergency COVID-19 hospitalizations from 369 hospitals
Exclude 3,768 and 0 hospital: transfer from another hospital
283,773 emergency COVID-19 hospitalizations from 369 hospitals
Exclude 6,342 and 4 hospitals: transfer to another acute care hospital
277,431 emergency COVID-19 hospitalizations from 365hospitals
Exclude 136,715 hospitalizations and 77 hospitals: not admitted to ICU
140,716 emergency COVID-19 hospitalizations admitted to ICU from 288 hospitals
Exclude 25 hospitalizations and 0 hospitals: include both present and former smoker ICD-10-CM codes
140,691 emergency COVID-19 hospitalizations admitted to ICU from 288 hospitals
Exclude 1,846 hospitalizations and 1 hospital: patient left against medical advice
138,845 emergency COVID-19 hospitalizations admitted to ICU from 287 hospitals
Exclude 0 hospitalizations and 0 hospitals: missing hospital disposition data
138,845 emergency COVID-19 hospitalizations admitted to ICU from 287 hospitals
22,893 former smoker emergency hospitalizations admitted to ICU from 259
9,157 current smoker emergency COVID-19 hospitalizations admitted to ICU from 249 hospitals
106,795 never-smoker emergency COVID-19 hospitalizations admitted to ICU from 282 hospitals

2. Potential Survivor Bias and “Smoker’s Paradox”

• The finding that smokers had lower in-hospital mortality contradicts prior literature suggesting that smoking is a risk factor for severe COVID-19 outcomes. This could be due to survivor bias, where:

o Sicker smokers (with severe chronic lung disease, cardiovascular disease) may have died before hospitalization, meaning only relatively healthier smokers were included in the dataset.

o More severe COVID-19 cases among smokers may have been less likely to make it to the hospital.

• Recommendation: The discussion should highlight and critically assess the potential survivor bias as a key limitation. If possible, authors should compare pre-hospital mortality rates among smokers and non-smokers (e.g., by referring to external epidemiological data).

We thank the reviewer for this important recommendation. Several steps have been taken to address this issue.

First, we have computed the E-value for unmeasured confounders to quantify the association on the risk-ratio scale that unmeasured confounders would have to have with both mortality and smoking (beyond the controlled for mediators and cofounders) in order to explain away the observed association. The E-value and potential unmeasured confounders are now directly shown within a new directed acyclic graph (Figure 1)

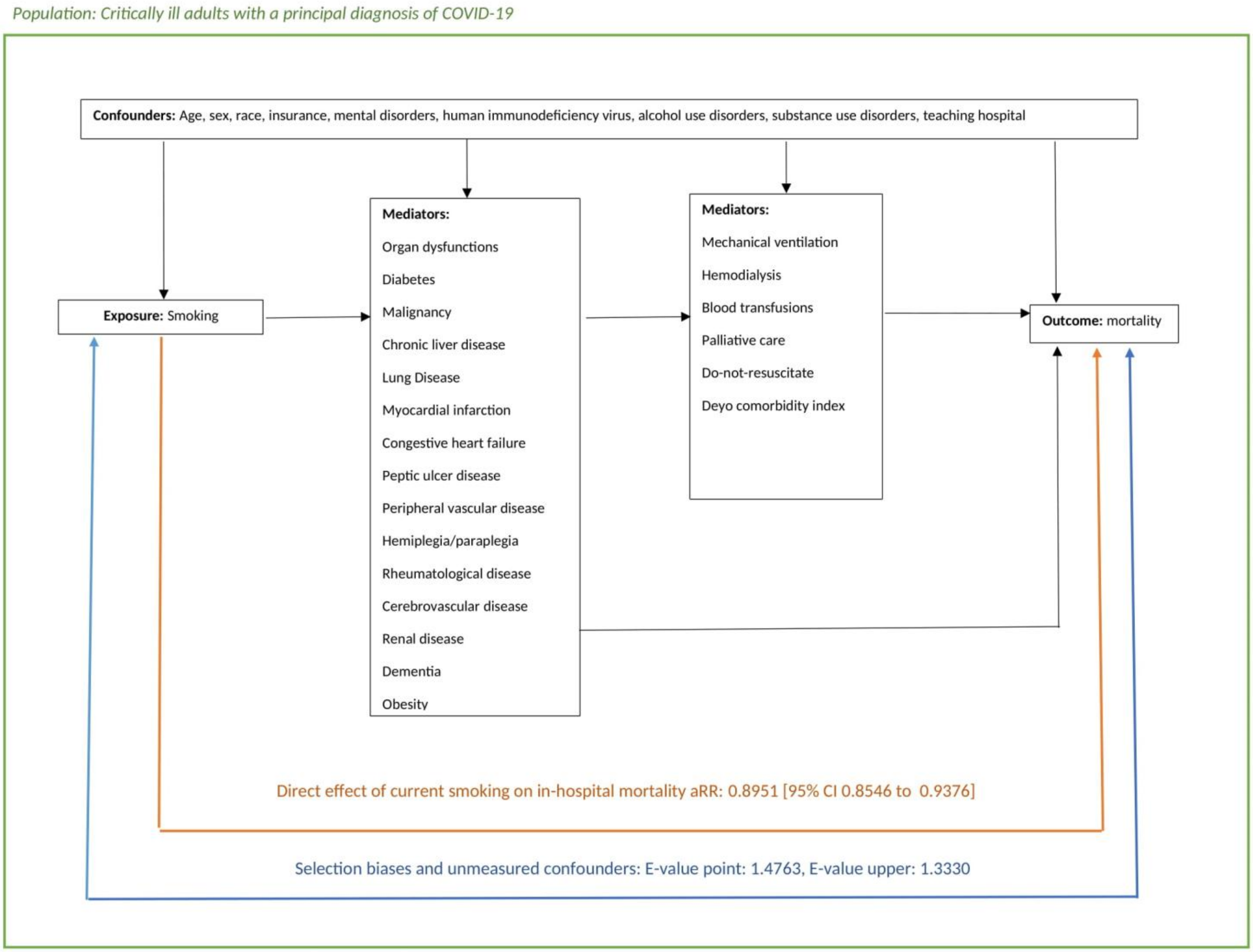

Secondly, we have created a directed acyclic graph (see new Figure 4) that is a causal diagram illustrating the situation under which selection on hospitalized patients admitted to the ICU could result in selection bias on severity of COVID-19 illness.

Adults with COVID-19

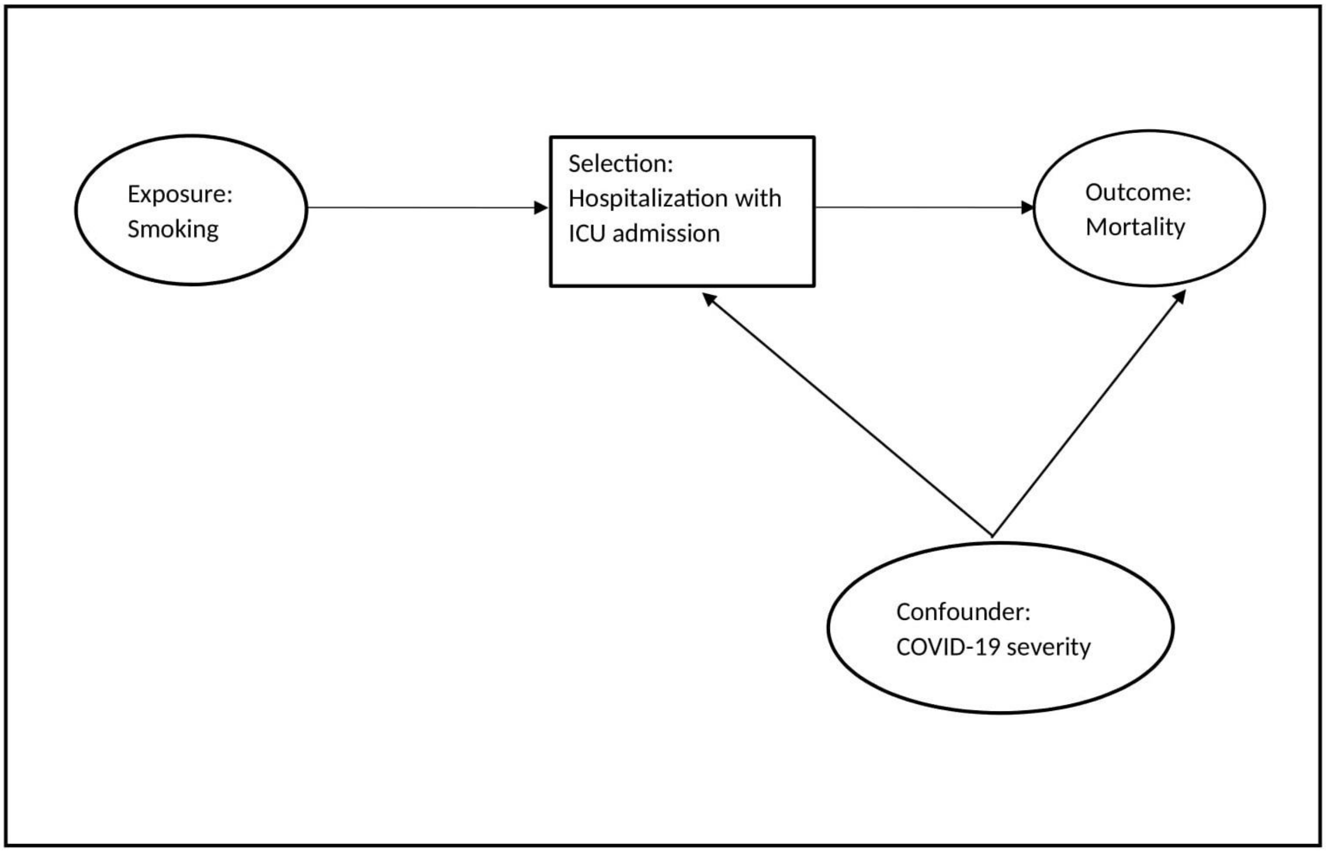

Third, to critically assess the potential of selection bias we added a new supplementary table shown below. If the reasoning that smokers die before arriving at the hospital is true and that this leads to lower hospital mortality rates then it is logical to assume that other known risk factors for mortality among COVID-19 or other substance use and mental disorders would demonstrate a similar pattern. However, smoking stands out among these risk factors as

having much lower in-hospital and short-term crude mortalities. None of the other examined risk factors demonstrate this pattern. bias or other selection bias in the cohort derivation or regression models.

S6 Table. Additional analyses for the evaluation of potential selection and collider biases

Exposure	Exposed	Unexposed	aRR [95% CI][a]	*p* value
	mortalities / total no. (%)			
**Current vs never smoking**				
In-hospital mortality	990 / 9,157 (10.8)	17,433 / 106,795 (16.3)	0.8951 [0.8546 to 0.9376]	< 0.0001
Short-term mortality	1,256 / 9,157 (13.7)	21,596 / 106,795 (20.2)	0.8988 [0.8664 to 0.9325]	< 0.0001
**Chronic lung disease**				
In-hospital mortality	3,446 / 22,752 (15.1)	14,977 / 93,200 (16.1)	0.9781 [0.9462 to 1.0110]	0.1896
Short-term mortality	4,577 / 22,752 (20.1)	18,275 / 93,200 (19.6)	0.9822 [0.9579 to 1.0070]	0.1575
**Congestive heart failure**				
In-hospital mortality	4,518 / 22,889 (19.7)	13,905 / 93,063 (14.9)	1.0079 [0.9614 to 1.0566]	0.7442
Short-term mortality	5,942 / 22,889 (26.0)	16,910 / 93,063 (18.2)	0.9870 [0.9524 to 1.0229]	0.4723
**Malignancy**				
In-hospital mortality	864 / 4,537 (19.0)	17,559 / 111,415 (15.8)	1.0006 [0.8788 to 1.1392]	0.9932
Short-term mortality	1,246 / 4,537 (27.5)	21,606 / 111,415 (19.4)	1.0595 [0.9662 to 1.1618]	0.2189
**Diabetes**				
In-hospital mortality	8,969 / 50,615 (17.7)	9,454 / 65,337 (14.5)	0.9943 [0.9740 to 1.0151]	0.5901
Short-term mortality	10,826 / 50,615 (21.4)	12,026 / 65,337 (18.4)	0.9791 [0.9631 to 0.9953]	0.0115
**Obesity**				
In-hospital mortality	6,761 / 39,215 (17.2)	11,662 / 76,737 (15.2)	1.0291 [1.0119 to 1.0466]	0.0008
Short-term mortality	7,584 / 39,215 (19.3)	15,268 / 76,737 (19.9)	1.0712 [1.0494 to 1.0935]	< 0.0001
**Alcohol abuse**				
In-hospital mortality	319 / 1,833 (17.4)	18,104 / 114,119 (15.9)	0.8853 [0.7954 to 0.9855]	0.0259
Short-term mortality	389 / 1,833 (21.2)	22,463 / 114,119 (19.7)	0.9099 [0.8373 to 0.9887]	0.0259

**Drug abuse**				
In-hospital mortality	350 / 2,183 (16.0)	18,073 / 113,769 (15.9)	0.8830 [0.8098 to 0.9629]	0.0048
Short-term mortality	410 / 2,183 (18.8)	22,442 / 113,769 (19.7)	0.8708 [0.8120 to 0.9338]	0.0001
**Mental disorders**				
In-hospital mortality	4,416 / 24,287 (18.2)	14,007 / 91,665 (15.3)	1.0006 [0.9780 to 1.0238]	0.9566
Short-term mortality	5,617 / 24,287 (23.1)	17,235 / 91,665 (18.8)	0.9986 [0.9812 to 1.0163]	0.8762

[a] aRR [95% CI]: Adjusted risk ratio and 95% confidence interval

Fourth, we have computed the selection bias bounding factor the primary analysis and added the following to the manuscript:

Sensitivity analyses for selection bias. *In the case of inference within a sampled population, the minimum level of selection bias, on the risk ratio scale, needed to explain away the observed direct effect of smoking on mortality have the same formulas as the E-value for unmeasured confounding [33]. Thus, if selection bias explains away the observed association, selection bias must have resulted in an unmeasured confounder that increases the risk of death by 1.3330 on the risk ratio scale and also differs between never smokers and current smokers by 1.3330 on the risk-ratio scale (each within the sample.)*

However, we have decided against using additional external epidemiological data as the manuscript is already long and complex. We request a pass on this issue.

3. Smoking Definition and Intensity

• Smoking status was defined using ICD-10-CM codes for current and former smokers (Table S1). However:

o How recent is "former smoking" considered?

o Was smoking intensity assessed (e.g., pack-years)? Prior literature has shown that smoking severity impacts disease outcomes.

• Recommendation: If possible, stratifying former smokers by time since cessation and current smokers by smoking intensity could provide more granular insights. Otherwise, the limitation of not differentiating smoking intensity should be discussed.

We thank the reviewer for this important observation. In response we have redefined the exposure variable to have three disjoint levels: never smokers, former smokers, and current smokers and updated the supplementary tables on smoking codes. ICD-10-codes for current and former smoking do not provide information on the smoking intensity. Although codes for current smoking could be further divided into smaller classifications like withdrawal, remission, etc, we believe this would add additional complexity to the analyses and would not change the final conclusions that there is a smoker's paradox. We request another pardon on this issue. However, we have now mentioned these issues in the limitations section. It is important to note these issues of intensity and timing in order to correctly interpret the model results, thank you.

4. Inconsistencies in ICU Admissions, Mechanical Ventilation, and Disease Severity

• Non-smokers had higher ICU admission rates (53.1% vs. 50.6%) and required more mechanical ventilation (9.7% vs. 7.1%), despite having a lower Deyo comorbidity index. This is counterintuitive, as one would expect more critically ill smokers requiring these interventions.

• Recommendation: The authors should explore potential reasons for this discrepancy.

o Were non-smokers admitted later in the disease course when they were already severely ill?

o Were hospital admission and treatment practices different for smokers vs. non-smokers?

o If this discrepancy was due to unmeasured confounders, this should be explicitly stated in the discussion.

We thank the reviewer for these important suggestions. In response we have made several changes.

First, we have revised the analytic cohort to only include those admitted to the ICU and only patients who were emergency admissions.

Second, the cohort characteristics subsection of the results section now mentions the difference in mechanical ventilation as a potential indicator of difference in illness severity.

Third, the methods and results section now explicitly mention three examples of possible selection bias that could result COVID-19 illness severity as a confounder.

Fourth, we have used propensity score overlap weighting to switch the target population to one at clinical *equipoise*. This results in exact balance on the weighted mean of all covariates within the resulting overlap population. Briefly, the overlap weighted population is a pseudo-population whose elements are those from the original population but whose frequencies are changed. Thus, imbalances in Deyo index, mechanical ventilation, and all other risk adjustment covariates including type of insurance have been eliminated. Note that g-computation has still allowed for risk adjustment on this overlap weighted population using logistic regression. We have also mentioned the inability to measure differences in illness severity and process of care that may be different between smokers and non-smokers.

Fifth, we have computed E-values for unmeasured confounders and have created a directed acyclic graph.

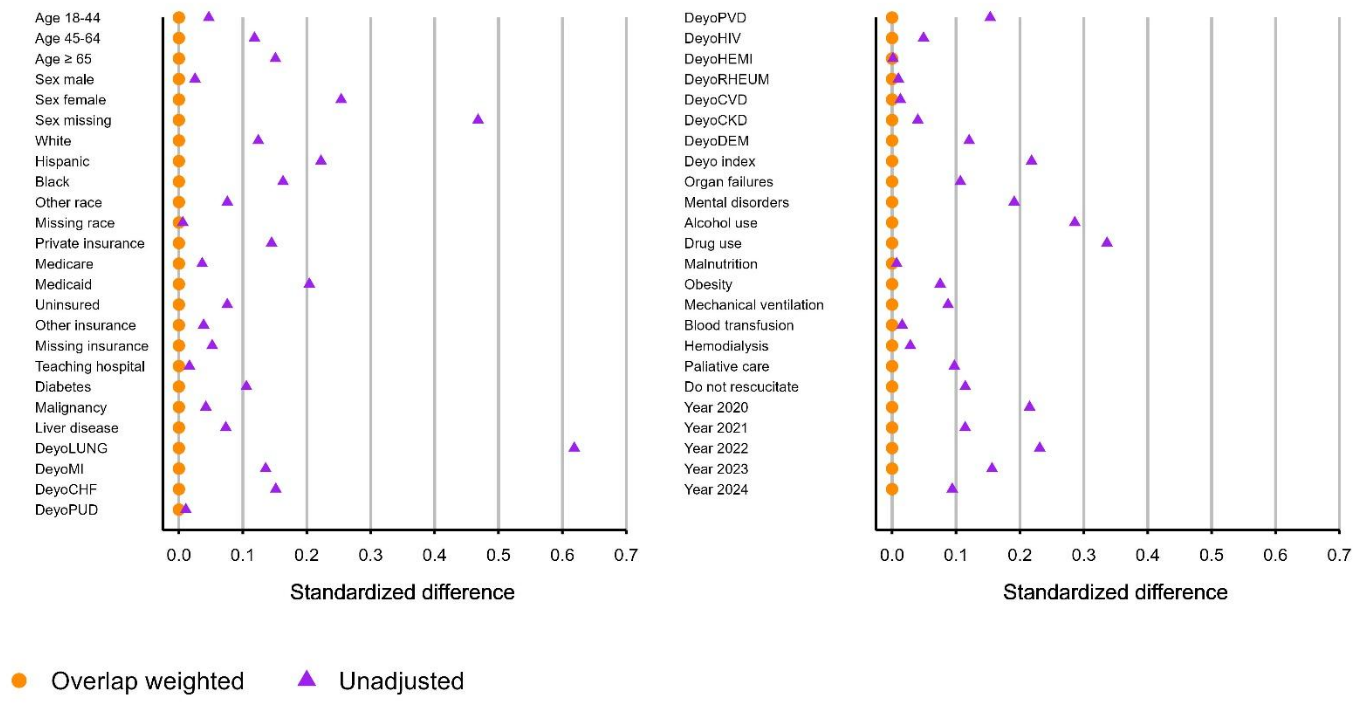

5. Model Complexity and Overfitting Risk

• The multivariable models included 11 covariates. While the dataset is large, including too many covariates in a logistic regression can introduce overfitting and multicollinearity issues.

We appreciate the reviewers' attention to this important topic.

• Recommendation:

o The authors assessed variance inflation factors (VIFs) for multicollinearity, its details should be shared.

Minor Concerns and Additional Recommendations:

We have provided a supplementary table providing the variance inflation factors for the new primary model. The table shows that only the Deyo comorbidity index has high generalized variance inflation factor which is to be expected since it is a linear combination of the Deyo comorbidities.

However, the high variance inflation factor does not automatically invalidate the model especially since the focal variable of smoking status does not have high variance inflation factor, but instead indicates that the model *could* be unstable. We have provided a supplementary table for alternative modeling for the potential impact of multicollinearity which demonstrates no material impact of multicollinearity on the model results.

S5 Table. Alternative modeling for the potential impact of multicolinearity				
**Model**	**Current smokers**	**Never smokers**	**aRR [95% CI][a]**	**aRD [95% CI][b]**
	mortalities total no. (%)			
**With Deyo comorbidity index**				
In-hospital mortality	990 / 9,157 (10.8)	17,433 / 106,795 (16.3)	0.8951 [0.8546 to 0.9376]	-0.0135 [-0.0190 to -0.0081]
Short-term mortality	1,256 / 9,157 (13.7)	21,596 / 106,795 (20.2)	0.8988 [0.8664 to 0.9325]	-0.0164 [-0.0219 to -0.0110]
**Without Deyo comorbidity index**				
In-hospital mortality	990 / 9,157 (10.8)	17,433 / 106,795 (16.3)	0.8957 [0.8552 to 0.9383]	-0.0134 [-0.0189 to -0.0080]
Short-term mortality	1,256 / 9,157 (13.7)	21,596 / 106,795 (20.2)	0.8997 [0.8672 to 0.9334]	-0.0163 [-0.0217 to -0.0108]

[a] aRR [95% CI]: Adjusted risk ratio and 95% confidence interval

[b] aRD [95% CI]: Adjusted risk difference and 95% confidence interval

6. Statistical Considerations

• Line 93-94: "Continuous variables are summarized as mean and standard deviation."

o Was normality tested before using mean (SD)? If data were non-normally distributed, median (IQR) should be reported instead.

The continuous variables, number of organ dysfunctions, length of stay, and Deyo comorbidity index are skewed right and they are truncated at zero on the left. They are non-normal as confirmed with a Shapiro-Wilk's test. To be careful and because we now have three levels of exposure we have used the permutational and nonparametric multivariate Welch t-test on distances to test for differences in the means of continuous variables. Also, just because a variable is not normally distributed does not imply that median and IQR are more appropriate. In the present case, deyo index and number of organ dysfunctions have the same median in each exposure group due to their truncation on left at zero and right skewed nature. If the median and IQR were to be reported it could confuse readers into thinking the distributions are the same when they are not. Thus, we have retained use of mean as it is a helpful summary in the current situation.

A review of textbooks on mathematical statistics also demonstrates that the mean is an important and useful summary statistic for several non-normal distributions including gamma, lognormal etc.

• Line 94: “Fisher’s test was used for comparison of categorical variables.”

o Why was Fisher’s test used instead of chi-square? Fisher’s test is typically used when expected cell counts are small (<5). If used for large categorical variables, a chi-square test would be more appropriate.

Originally, we used Fisher’s test because it is an exact test and represents the true underlying process under the null hypothesis of no difference in population proportions. Thus, Fisher’s test is more natural and interpretable capturing the underlying process given the null hypothesis. However, we have still switched to the chi-square test per your suggestion as it may be more familiar to readers.

7. Smoking and Hospital Disposition

• More smokers left against medical advice (LAMA) (2.4% vs. 1.2%).

o How were these patients accounted for? Could their outcomes have differed from hospitalized patients? It should be noted in the discussion.

We have extended the cohort to exclude hospitalization with against medical advice discharges.

• Insurance Data Missing for More Non-Smokers.

o Was there a difference in healthcare access? Did this lead to delayed hospital presentation or differences in treatment? This potential confounder should be acknowledged.

We have also mentioned in the limitations section the inability to measure differences in illness severity and process of care that may be different between smokers and non-smokers. The key findings section of the discussion now reviews a possible socio-economic selection bias. In addition, we have computed E-values for unmeasured confounders and have created a directed acyclic graph. The use of the overlap weighted population means that differences in missing insurance between exposure strata have been eliminated as have differences in rates of medical procedures. In particular, current and never smokers have the same rates of hemodialysis, invasive mechanical ventilation, blood transfusions, palliative care, and do not resuscitate orders. (See table two below)

Table 2. Overlap weighted means of COVID-19 hospitalizations for current and never smokers

Variables	Never smokers[a]	Current smokers[a]
	P = 0.5000	P = 0.5000
**Age, years (%)**		
18-44	17.6	17.6
45-64	40.3	40.3
≥ 65	42.1	42.1
**Gender (%)**		
Male	50.2	50.2
Female	37.7	37.7
Suppressed	12.1	12.1
**Race/ethnicity (%)**		
White	49.9	49.9
Hispanic	26.7	26.7
Black	17.1	17.1
Other Race	6.2	6.2
Missing race	0.0	0.0
**Insurance (%)**		
Private insurance	44.2	44.2
Medicare	29.8	29.8
Medicaid	7.9	7.9
Uninsured	9.2	9.2
Other insurance	6.6	6.6
Missing insurance	2.3	2.3
**Deyo index (mean)**	1.71	1.71
**Deyo-Charlson comorbidities (%)**		
Diabetes	40.2	40.2
Malignancy	4.5	4.5

Chronic liver disease	5.5	5.5
Lung disease	40.2	40.2
MI	11.6	11.6
CHF	24.1	24.1
Peptic ulcer disease	0.7	0.7
Peripheral vascular disease	4.1	4.1
HIV	0.4	0.4
Hemiplegia/paraplegia	1.1	1.1
Rheumatological diseases	3.1	3.1
Cerebrovascular disease	4.3	4.3
Renal disease	19.1	19.1
Dementia	5.8	5.8
**Other comorbidities (%)**		
Mental disorders	27.0	27.0
Alcohol use disorders	4.6	4.6
Drug use disorders	5.5	5.5
Malnutrition	8.3	8.3
Obesity	31.9	31.9
**Medical procedures (%)**		
Mechanical ventilation	10.0	10.0
Blood transfusions	2.9	2.9
Hemodialysis	4.5	4.5
Palliative care	6.5	6.5
Do not resuscitate	12.6	12.6
**Year of discharge (%)**		
Year 2020	22.8	22.8
Year 2021	41.3	41.3
Year 2022	23.2	23.2

Year 2023	9.2	9.2
Year 2024	3.5	3.5
**Teaching hospital (%)**	21.3	21.3
**Organ dysfunctions (mean)**	1.51	1.51
**Mortality**		
In-hospital (%)	12.8	11.5
Short-term (%)	16.1	14.4

[a] Percentages may not add to 100.0 due to rounding

8. Limitations of the TIPUDF Database

• The authors acknowledge that TIPUDF does not provide illness severity data or detailed treatment information (e.g., oxygen therapy, corticosteroid use).

• Recommendation: Expand on this limitation in the discussion. The lack of disease severity data could lead to residual confounding, as smokers and non-smokers may have had different baseline COVID-19 severity that was not captured in the dataset.

We thank the reviewer for this helpful suggestion. We have extended the limitations section to include this.

Also, the methods and results sections have been significantly expanded to review, quantify, and visualize potential selection biases that could result in COVID-19 illness severity as a confounder. These additions include the below image showing hospitalization as a collider and illness severity as confounder.

Adults with COVID-19

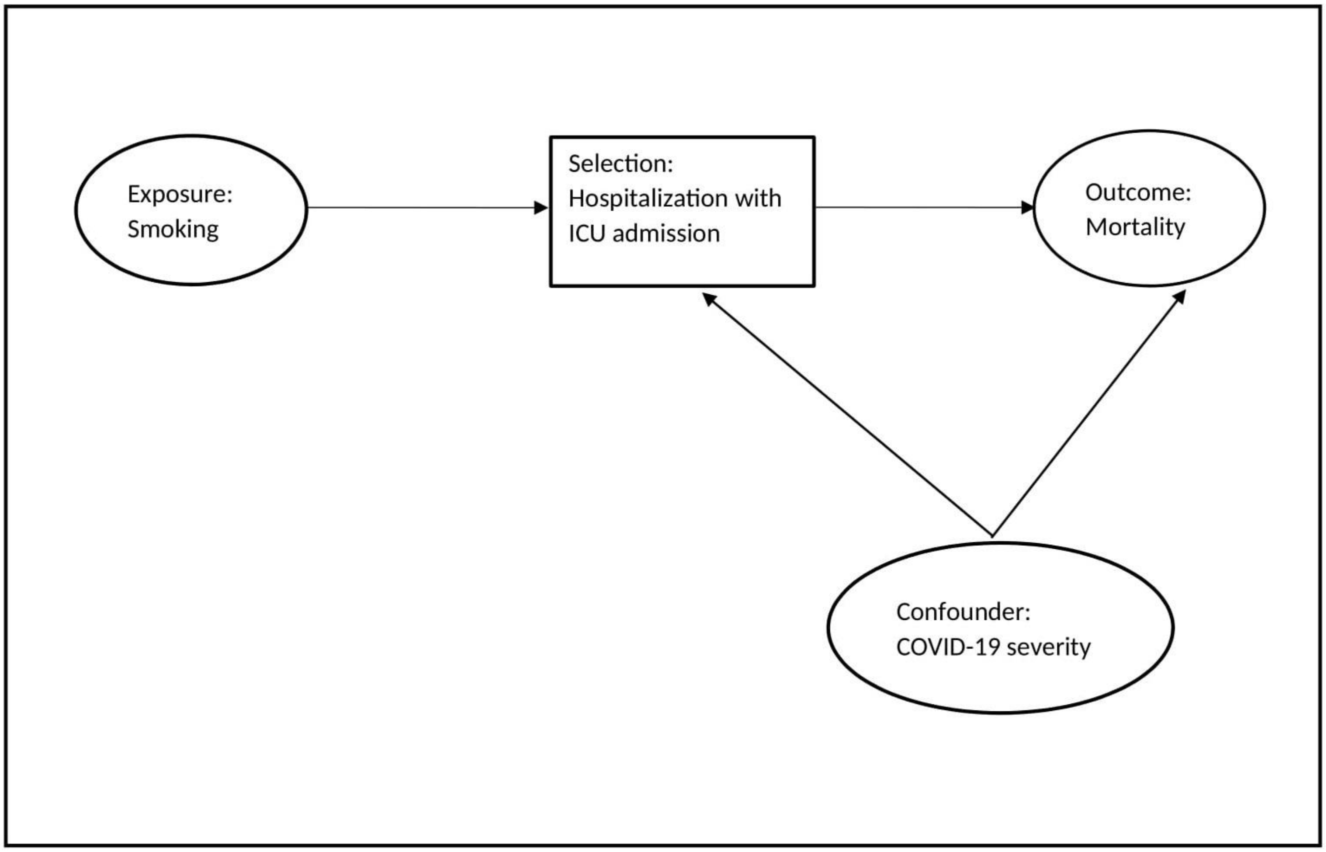

9. Discussion of Prior Studies

• The discussion compares findings to multiple prior studies but does not critically assess key methodological differences in enough detail.

We have added discussion about the use overlap weights and also enhanced the review of some articles

o Line 257: "The tenfold difference in smoking between our study and theirs suggests that smoking was defined and identified differently."

♣ What was their definition, and how does it differ?

The recently published Brazilian study combined current and former smokers into a single class of smokers using a self-report column of their data base. This differs from our study where we have separated current and former smokers from each other and used ICD-10-CM codes.

o Line 225-229: "Many prior studies only included hospitalizations during the early pandemic (2019-2020)."

♣ What are the implications? Earlier in the pandemic, ICU capacity was more limited, and treatment protocols were different.

♣ Did later treatment strategies (e.g., corticosteroids, antivirals) modify the effect of smoking on mortality?

We have added a new table for sensitivity analyses by year of discharge that demonstrates a four-fold reduction in mortality rates of COVID-19 patients since 2020. This table may assist in explaining the importance of our study including more recent years. Results obtained at the beginning of the pandemic may no longer be valid in later years. An important question that may be asked of previous studies is the following: If the study was replicated today would the results be same as were found in 2020? Because, mortality rates have changed so much, there is reason to question. The study of modified effects of antivirals, corticosteroids, and other treatments is important but we believe that is not central to our study and although important and interesting is best explored in a separate study. We have added to the discussion by mentioning this important possibility,

Table 5. Sensitivity analyses by year for the association of smoking status and mortality among critically-ill COVID-19 patients

**Subgroup**	**Current smokers**	**Never smokers**	**aRR [95% CI][a]**	**aRR [95% CI][a]**	***p* value**
	mortalities / total no. (%)				
**2020[c]**					
In-hospital mortality	247 / 1,960 (12.6)	5,363 / 32,893 (16.3)	0.9285 [0.8491 to 1.0152]	-0.0101 [-0.0219 to 0.0017]	0.1032
Short-term mortality	291 / 1,960 (14.8)	6,502 / 32,893 (19.8)	0.9154 [0.8497 to 0.9861]	-0.0142 [-0.0258 to -0.0027]	0.0198
**2021**					
In-hospital mortality	547 / 3,650 (15.0)	9,551 / 48,600 (19.7)	0.9209 [0.8689 to 0.9760]	-0.0134 [-0.0226 to -0.0043]	0.0054
Short-term mortality	648 / 3,650 (17.8)	11,208 / 48,600 (23.1)	0.9297 [0.8870 to 0.9745]	-0.0140 [-0.0228 to -0.0052]	0.0024
**2022**					
In-hospital mortality	158 / 2,287 (6.9)	2,094 / 16,801 (12.5)	0.8011 [0.7014 to 0.9150]	-0.0188 [-0.0293 to -0.0083]	0.0011
Short-term mortality	238 / 2,287 (10.4)	3,001 / 16,801 (17.9)	0.8103 [0.7378 to 0.8899]	-0.0263 [-0.0373 to -0.0153]	< 0.0001

**2023**					
In-hospital mortality	28 / 914 (3.1)	315 / 6,174 (5.1)	0.6510 [0.4661 to 0.9093]	-0.0170 [-0.0286 to -0.0053]	0.0118
Short-term mortality	58 / 914 (6.3)	635 / 6,174 (10.3)	0.7840 [0.6307 to 0.9745]	-0.0187 [-0.0343 to -0.0031]	0.0283
**2024**[d]					
In-hospital mortality	10 / 346 (2.9)	110 / 2,327 (4.7)	1.0418 [0.6106 to 1.7774]	0.0013 [-0.0157 to 0.0183]	0.8806
Short-term mortality	21 / 346 (6.1)	250 / 2,327 (10.7)	0.8416 [0.5889 to 1.2025]	-0.0125 [-0.0374 to 0.0123]	0.3435

[a] aRR [95% CI]: adjusted risk ratio and 95% confidence interval

[c] Q2, Q3, Q4 only

[d] Q1, Q2 only

Reviewer #4: Changes looks good

-add about the deyo comorbidity index and how did you calculate in the methodology section

We have mentioned in the new subsection on variance inflation factors that the Deyo index is a linear combination of the Deyo comorbidities. Also, the references can assist readers.

-Page 14: Restructure the sentence 'Thus, the association of smoking and mortality begins to fade away as the health of COVID-19 patients deteriorate' as this is the result based on your analysis and not a established fact. You can say in simple words that our analysis show diminishing association between smoking and mortality among severe covid-19 patients.

We thank the reviewer for this important observation. As a result of our new analyses, that sentence is no longer included.

-your limitations should include a possibility of reporting bias among patients regarding their smoking habits. it should also include that both former and current smokers were grouped into one exposure categories. they were not analyzed as compared to previous studies.

We have changed our exposure to have three levels as in prior studies: never, former, and current smokers.
