# Supplementary material for: Nicotine dependence among critically ill COVID-19 patients: A population-based cohort study
Source: PLoS One. 2026 Apr 22;21(4):e0308776. doi: 10.1371/journal.pone.0308776 (PMC13102216; doi:10.1371/journal.pone.0308776)
Supplement: Attachment — Submitted filename: Response_to_Reviewers_auresp_4.pdf [file pone.0308776.s014.pdf]

November 10, 2025

Dear PLOS ONE,

We appreciate the editor comment and the positive comments by the reviewer. We have adjusted the manuscript by deleting the sentence that is ambiguous and confusing. Note that the same information is available in the cohort derivation diagram where the meaning is clearer in that it leads to a partition of hospitalizations with three mutually exclusive groups; never smokers, former smokers, and current smokers.

Sincerely,

John Garza

John Garza, PhD

Associate Professor

Texas Tech University School of Medicine

gar31703@ttuhsc.edu

512-350-1444

- Additional Editor Comments:

The methods section currently states that "hospitalizations with a diagnosis of both former and current smoking were excluded from the study (n = 25)". Considering that the authors compared never smokers with current and former smokers, it is unclear what the authors meant to indicate here. Please clarify.

In our effort to divide hospitalizations into never smokers, former smokers, and current smokers, we used ICD-10-CM codes as presented in the Supplementary Materials. The reason for this sentence relates to information found on the American Lung Association Billing Guide for Tobacco Screening and Cessation 2021 (linked below)

https://www.lung.org/getmedia/275e15df-413d-450f-9bed-b98a9fb04e1a/ala-billing-guide-2021.pdf

The information found on pages nine and thirteen indicate that ICD-10-CM code Z87891 should not be used together with ICD-10-CM F17 codes. Thus, each hospitalization should either have only Z87891 and not F17 codes, have a F17 code but not Z87891, or not have Z87891 and not have F17 codes. This was mostly observed in our cohort derivation except for a small number (n=25) and we decided that we could not determine the correct smoking status of these hospitalizations. They were therefore excluded.

Billing Guide for Tobacco Screening and Cessation

Diagnosis Coding Guide

ICD-10 Diagnosis Code	Description: All with Nicotine Dependence
**Z Codes**	
Z57.31	Occupational exposure to environmental tobacco smoke<br>• May not be used with Z77.22 exposure to environmental smoke.
Z77.22	Contact with and suspected exposure to environmental smoke<br>• May not be used with a F17.2 tobacco dependence or Z72 tobacco use code.
Z71.6	Counseling and Medical Advice – tobacco abuse counseling
Z72.0	Problems Related to Lifestyle and tobacco use not otherwise specified
Z87.891	Personal history of nicotine dependence<br>• May not be used with F17.2 current nicotine dependence code.
Z13.89	Encounter for screening for other disorder. Use for tobacco use screening.
**Maternal Tobacco Use and Newborn Exposure**<br>• For maternal use add an additional F17 code to indicate type of tobacco	
O99.330	Smoking (tobacco) complicating pregnancy, unspecified trimester
O99.331	Smoking (tobacco) complicating pregnancy, first trimester
O99.332	Smoking (tobacco) complicating pregnancy, second trimester
O99.333	Smoking (tobacco) complicating pregnancy, third trimester
O99.334	Smoking (tobacco) complicating childbirth
O99.335	Smoking (tobacco) complicating the puerperium
P04.2	Newborn suspected to be affected by exposure in utero to tobacco smoke May not be used with P96.81 newborn exposure to environmental tobacco smoke.
P96.81	Exposure to (parental) (environmental) tobacco smoke in the perinatal period.

Documentation

Regardless of the payer (e.g. Medicare, Medicaid, private), providers need to use ICD-10 codes and provide documentation regarding medical necessity and the specifics of what was provided. The goal is to clearly establish medical necessity and ensure payment for services. **Coding is not sufficient**. Medicare and other payers find improper payments by selecting a sample of claims or flagging suspicious claims and requesting medical documentation from the provider. The claim is reviewed against the provider's medical documentation – either an electronic medical record or paper record. As such, the following items should be documented in the medical record:

- Patient's willingness to attempt to quit
- What was discussed during counseling
- Amount of time spent counseling
- Tobacco use
- Advice to quit and impact of smoking provided to patient
- Methods and skills suggested to support cessation
- Medication management
- Setting a quit date with the patient
- Follow-up arranged
- Resources made available to the patient

Documentation Tips

- The F17 codes are used if the patient is dependent on tobacco. The Z codes are used if there is NOT dependence on tobacco. The Z codes cannot be combined with an F17 code.
- Using the term "history of" or "personal history of" means a past medical condition that no longer exists. If used for a current condition, payment will be denied.
  - History of may be an appropriate reason to use the Z code.
- Documentation must include a treatment plan for each diagnosis (e.g. refer to oncologist) and an assessment, such as "stable," "worsening," "not responding to treatment."
- Use linking terms to connect the diagnoses and manifestations, such as "due to" or "secondary to."
- Behavioral health providers are qualified to use behavioral or mental health diagnoses such as F17.200 as the primary rationale for their services. Medical providers may not and so must select a diagnosis code that accurately reflects the biological impact of tobacco use such as one of the T65.2 options.
- Be sure to document "counseling" activities (advising about specific changes to routines, arranging [illegible]

We agree with the editor's assessment that the sentence in unclear and have chosen to delete this sentence from the manuscript. Figure 2 however still communicates this fact but it is clearer from the

figure what is intended and the diagram ends in nonoverlapping groups. We respectfully request that the editor accept our proposed resolution.

6. Review Comments to the Author

Reviewer #3: The authors have addressed the core methodological concerns with substantial and well-reasoned revisions: redefining the cohort to principal COVID-19 diagnosis, excluding AMA discharges, expanding limitations, separating smoking exposure into never/former/current, adding quantitative bias analyses, implementing propensity score overlap weighting with balance diagnostics, reporting VIFs with an alternate model (w/ and w/o Deyo), switching to χ^2 for large-sample categorical comparisons, and providing year-stratified sensitivity analyses. The additions (revised flow diagram, DAGs, overlap-weighted balance table) meaningfully improve internal validity and transparency.

We thank the reviewer for the many helpful comments and the time invested in carefully checking the many details of the study.
