# Supplementary material for: Nicotine dependence among critically ill COVID-19 patients: A population-based cohort study
Source: PLoS One. 2026 Apr 22;21(4):e0308776. doi: 10.1371/journal.pone.0308776 (PMC13102216; doi:10.1371/journal.pone.0308776)
Supplement: Attachment — Submitted filename: Response_to_Reviewers_auresp_5.pdf [file pone.0308776.s015.pdf]

Dear PLOSONE,

Thank you for the insightful comments and the opportunity to revise the manuscript. In response to the editor's valuable suggestions, we have made adjustments to the manuscript. After studying the *ICD-10-CM Tabular List of Diseases and Injuries,* the *Clinical Classification Software Refined for ICD-10-CM Diagnosis*, and the *FY 2024 ICD-10-CM Official Guidelines for Coding and Reporting*, we realized that both "smoking" and "nicotine user" are not well defined or clearly identifiable within ICD-10-CM. As a result, we have changed the exposure to be "nicotine dependence" which, in contrast, is well defined in ICD-10-CM. The methods and structure of the manuscript are the same as before and the results are practically the same as before. Key adjustments to the manuscript, intended to improve technical accuracy, assist readers in interpreting the results, and assist in evaluating the study, include

1. A revised Table S1 that using the editor's guidance for ICD-10-CM definition of currently and formerly nicotine dependent individuals
2. A new Table S2 in the supplementary materials file that lists each individual ICD-10-CM code for nicotine dependence along with the code description and prevalence within the cohort
3. A new Table S6 in the supplementary materials file that provides a sensitivity analysis for the class of nicotine dependence including for nicotine dependence cigarettes
4. A comprehensive review of ICD-10-CM codes for smoking and nicotine user *vs* nicotine dependence in the exposure and outcome subsection of the materials and methods section
5. A revised and improved Figure 1 (Directed acyclic graph)
6. Expansion of the study cohort to include Q3 and Q4 of 2024 as this data was now available
7. Additional R code in the supplementary materials file demonstrating properties of clinical *equipoise* in the overlap weighted population
8. Additional R code in the supplementary materials file comparing the confidence intervals obtained through nonparametric bootstraps to those obtained using the primary analysis procedure.
9. Throughout the manuscript "smoker" has been replaced by "nicotine dependent individual"
10. We have added information on the effective sample size of the overlap weighted population

==============================

The critical issue is that it appears that incorrect codes have been used. Please see the comments from the editor below and clarify, correct, or edit as appropriate. Please note that the analyses may be performed again using a different set of patients according to the correct codes or the conclusions (from the title on) must be thoroughly edited to clearly indicated that the population analyzed was nicotine users, not necessarily smokers.

==============================

Additional Editor Comments :

Thank you for addressing all previous comments. The manuscript has now been reviewed by two additional experts.

We appreciate the time invested by the additional reviewers.

From the description provided in the supplementary materials and methods, it appears that individuals were classified as current smokers using the following codings:

F17200, F17201, F17203, F17208, F17209, F17210, F17211, F17213, F17218, and F17219.

However, all codes F1720X are used for Nicotine addiction, which includes smokers, chewing tobacco users, vaping, and nicotine gum or patch users.

Considering that there are specific codes referencing to smokers, the use of F1720X strongly suggests other types of addictions.

Only F1721X codes refer to smokers exclusively.

Moreover, F17211 (and F17201) refer to people in remission and are not to be used for current smokers.

Please clarify if the coding used was different from what appears to be described in the methods section. If the coding used was as described, though, the results must be re-analyzed using only the correct codes, or the conclusions (from the title on) must be changed from smokers/smoking to nicotine users / addiction and the inclusion of non-smokers must be clearly described.

If F17211 or F17201 was used for current smokers/ nicotine users, the data will need to be re-evaluated or the conclusions from the tile on must be changed to current or in remission nicotine users/smokers

Thank you for the insightful comments and the opportunity to revise the manuscript. In response to the editor's valuable suggestions, we have made adjustments to the manuscript. After studying the *ICD-10-CM Tabular List of Diseases and Injuries,* the *Clinical Classification Software Refined for ICD-10-CM Diagnosis*, and the *FY 2024 ICD-10-CM Official Guidelines for Coding and Reporting*, we realized that both "smoking" and "nicotine user" are not well defined or clearly identifiable within ICD-10-CM. As a result, we have changed the exposure to be "nicotine dependence" which, in contrast, is well defined in ICD-10-CM. The methods and structure of the manuscript are the same as before and the results are practically the same as before. Key adjustments to the manuscript, intended to improve technical accuracy, assist readers in interpreting the results, and assist in evaluating the study, include

1. A revised Table S1 that using the editor's guidance for ICD-10-CM definition of currently and formerly nicotine dependent individuals
2. A new Table S2 in the supplementary materials file that lists each individual ICD-10-CM code for nicotine dependence along with the code description and prevalence within the cohort
3. A new Table S6 in the supplementary materials file that provides a sensitivity analysis for the class of nicotine dependence including for nicotine dependence cigarettes
4. A comprehensive review of ICD-10-CM codes for smoking and nicotine user *vs* nicotine dependence in the exposure and outcome subsection of the materials and methods section
5. A revised and improved Figure 1 (Directed acyclic graph)
6. Expansion of the study cohort to include Q3 and Q4 of 2024 as this data was now available

7. Additional R code in the supplementary materials file demonstrating properties of clinical *equipoise* in the overlap weighted population
8. Additional R code in the supplementary materials file comparing the confidence intervals obtained through nonparametric bootstraps to those obtained using the primary analysis procedure.
9. Throughout the manuscript “smoker” has been replaced by “nicotine dependent individual”
10. We have added information on the effective sample size of the overlap weighted population

Sincerely,

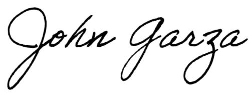

John Garza, PhD

Associate Professor

Texas Tech University School of Medicine
