# Supplementary material for: Nicotine dependence among critically ill COVID-19 patients: A population-based cohort study
Source: PLoS One. 2026 Apr 22;21(4):e0308776. doi: 10.1371/journal.pone.0308776 (PMC13102216; doi:10.1371/journal.pone.0308776)
Supplement: Attachment — Submitted filename: Response_to_Reviewers_auresp_6.pdf [file pone.0308776.s016.pdf]

March 11, 2026

Dear PLOSONE,

Thank you for the helpful editor comments. We have revised the manuscript and believe the revisions increase the accuracy and interpretability of the study. The title of the manuscript has been changed to “Nicotine dependence among critically ill patients with COVID-19: A population-based cohort study.” We have added an additional paragraph in the introduction section emphasizing that smoking and nicotine dependence are not subsets of each other preventing a direct comparison of this study to others using smoking as the exposure. A point-by-point response to the reviewer comments is provided below.

Thank you for submitting the revised manuscript, which has been enriched by the discussion about the potential confounders in the classification of patients. On reading the revised manuscript, it is not obvious why the title still includes the world "paradoxical". The manuscript makes it clear now that this study properly classifies the patients, which makes it difficult to compare to other published data. Not sure any adjective is needed, but "unpredicted" appears more consistent with the actual manuscript if one is to be used.

- Thank you for this insightful comment. We have changed the title to “Nicotine dependence among critically ill COVID-19 patients: A population-based cohort study**”**

The authors may want to consider including a brief discussion that the classification used does not include all smokers, many of whom are not dependent/addict to nicotine. It would help raise the awareness of the readers of any article analyzing the effects of "smoking" or nicotine addiction/dependence, or even use on any morbidity.

- Thank you for this comment. We have added the below paragraph to the introduction emphasizing the distinction between smoking and nicotine dependence.

“Smoking as an exposure does not require individuals to be dependent on nicotine and smoking includes non-tobacco substances such as cannabis that do not contain nicotine while nicotine dependence includes other forms of nicotine besides smoking or tobacco products. As a result, despite the intersection between smoking and nicotine dependence, they are different exposures with neither being a subset of the other. Smoking and nicotine dependence should be recognized as distinct variables for the purpose of scientific research. This complicates direct comparisons of studies using smoking as an exposure with those using nicotine dependence as an exposure in studies of COVID-19 or other diseases.”

Sincerely,

John Garza, PhD

Associate Professor

Texas Tech University School of Medicine

gar31703@ttuhsc.edu
